# Aptamer selection and applications for breast cancer diagnostics and therapy

**DOI:** 10.1186/s12951-017-0311-4

**Published:** 2017-11-13

**Authors:** Mei Liu, Xiaocheng Yu, Zhu Chen, Tong Yang, Dandan Yang, Qianqian Liu, Keke Du, Bo Li, Zhifei Wang, Song Li, Yan Deng, Nongyue He

**Affiliations:** 1 School of Chemistry and Chemical Engineering, Southeast University, Nanjing, 211189 People’s Republic of China; 2 State Key Laboratory of Bioelectronics, School of Biological Science and Medical Engineering, Southeast University, Nanjing, 210096 People’s Republic of China; 3 Economical Forest Cultivation and Utilization of 2011 Collaborative Innovation Center in Hunan Province, Hunan Key Laboratory of Green Chemistry and Application of Biological Nanotechnology, Hunan University of Technology, Zhuzhou, 412007 People’s Republic of China

**Keywords:** Aptamer, SELEX, Breast cancer, Diagnosis, Targeted therapy

## Abstract

Aptamers are short non-coding, single-stranded oligonucleotides (RNA or DNA) developed through Systematic Evolution of Ligands by Exponential enrichment (SELEX) in vitro. Similar to antibodies, aptamers can bind to specific targets with high affinity, and are considered promising therapeutic agents as they have several advantages over antibodies, including high specificity, stability, and non-immunogenicity. Furthermore, aptamers can be produced at a low cost and easily modified, and are, therefore, called “chemical antibodies”. In the past years, a variety of aptamers specifically bound to both breast cancer biomarkers and cells had been selected. Besides, taking advantage of nanomaterials, there were a number of aptamer-nanomaterial conjugates been developed and widely investigated for diagnostics and targeted therapy of breast cancer. In this short review, we first present a systematical review of various aptamer selection methods. Then, various aptamer-based diagnostic and therapeutic strategies of breast cancer were provided. Finally, the current problems, challenges, and future perspectives in the field were thoroughly discussed.

## Background

Breast cancer is currently the second leading cause of death among women in the world [1]. Although the mortality of breast cancer has decreased owing to the development of modern therapeutic approaches, the morbidity is still very high [2]. About 1,700,000 new cases of breast cancer and 521,900 deaths every year have been reported worldwide [3]. In China, especially in rural areas where people have lower income and less access to health care facilities, the morbidity of breast cancer has reached 24.20 per 100,000 [4]. In the United States, 231,840 women were diagnosed with and 40,290 died from breast cancer in 2015 [5]. As a highly heterogeneous malignancy, breast cancer can be classified into four subtypes based on the expression of specific biomarkers estrogen receptor (ER), progesterone receptor (PR), human epidermal growth factor receptor-2 (HER2), and proliferation marker Ki-67. That are luminal A (ER+ or PR+, HER2−), luminal B (ER+ or PR+, HER2+), HER2-overexpressing, and triple-negative (ER−, PR−, HER2−), whereas Ki-67 expression marks early-relapsing tumors [6–9]. In clinics, breast cancer subtypes usually show different response to therapy; therefore, treatment strategies are always chosen based on the molecular classification of tumors [10]. Early diagnosis and timely therapy are of a paramount importance in breast cancer prognosis, as the chances of survival significantly increase. However, current diagnostic technologies, including imaging, molecular detection, and immunohistochemistry (IHC) have inherent limitations and may provide uncertain results [11, 12]. The most common treatment strategies today are endocrine therapy, chemotherapy, and targeted therapy using antibodies recognizing cancer biomarkers; however, they are not always successful and may cause adverse effects and drug tolerance [13]. Therefore, there is a constant need to develop novel effective methods for breast cancer diagnosis and therapy.

Aptamers provide a potential alternative source of molecules for targeted therapy. Aptamers represent functional short single-stranded non-coding RNA or DNA that can specifically bind to targets with high affinity [14–20]; they are developed by the SELEX (Systematic Evolution of Ligands by EXponential enrichment) method in vitro and can recognize various targeting molecules by forming several secondary structures such as stem, loop, hairpin, bulge, pseudoknot, and G-quadruplex [21]. Compared with antibodies, aptamers have such advantages as high specificity and affinity, stability, and non-immunogenicity. In addition, they can be easily modified at a low cost to target a variety of molecules, and are, therefore, called “chemical antibodies” [22–25]. Because of these characteristics, in the past decade coupling with various nanomaterials, aptamers have been extensively studied in the fields of biological detection [26–30], molecular diagnosis [31, 32], bioimaging [33–35], tumor molecular classification [36], and drug delivery [37, 38].

Recently, applications of aptamers and aptamer-nanomaterial conjugates for cancer detection and therapy have attracted increasing attention. Aptamers with affinity to tumor molecular biomarkers are widely used for the detection of cancer markers and circulating tumor cells (CTCs), providing timely diagnosis, prognosis monitoring, and targeted therapy. Thus, a series of aptamers optimized for breast cancer have been selected and applied in diagnostics and treatment (Table 1), opening a new avenue for personalized medicine of breast cancer. A previously published review about aptamer-based diagnostics and targeted therapy in breast cancer focused on aptamer applications [39]; however, systematic analysis of aptamer selection methods has not yet been performed. Here, we evaluated various SELEX methods, discuss the applications of aptamers in diagnostics and treatment of breast cancer, and provide an outlook of current problems and future perspectives in this field.

**Table 1:** Aptamers specific for breast cancer

Aptamer	Sequence (5′–3′)	Target	References
H2	GGGCCGTCGAACACGAGCATGGTGCGTGGACCTAGGATGACCTGAGTACTGTCC	HER2	[40]
AS1411	GGTGGTGGTGGTTGTGGTGGTGGTGG	Nucleolin	[41, 42]
ERaptD4	ATACCAGCTTATTCAATTCGTTGCATTTAGGTG CATTACGGGGGTTATCCGCTCTCTCAGATAGTA TGTGCAATCA	ERα	[43]
S6	TGGATGGGGAGATCCGTTGAGTAAGCGGGCGTGTCTCTCTGCCGCCTTGCTATGGGG	SK-BR-3 cells	[44]
XLX-1-A	GAATTCAGTCGGACAGCGAAGTAGTTTTCCTT CTAACCTAAGAACCCGCGGCAGTTTAATGTAG ATGGACGAA	MDA-MB-231 cells	[45]
MUC1	GCAGTTGATCCTTTGGATACCCTGG	Mucin-1	[46]
HB5	AACCGCCCAAATCCCTAAGAGTCTGCACTTGT CATTTTGTATATGTATTTGGTTTTTGGCTCTCAC AGACACACTACACACGCACA	HER2	[47]
KMF2-1a	AGGCGGCAGTGTCAGAGTGAATAGGGGATGTA CAGGTCTGCACCCACTCGAGGAGTGACTGAGCGACGAAGACCCC	MCF-10AT1 cells	[48]
SYL3C	CACTACAGAGGTTGCGTCTGTCCCACGTTGTCATGGGGGGTTGGCCTG	EpCAM	[49]

## Selection of specific aptamers using SELEX

**Fig. 1:**
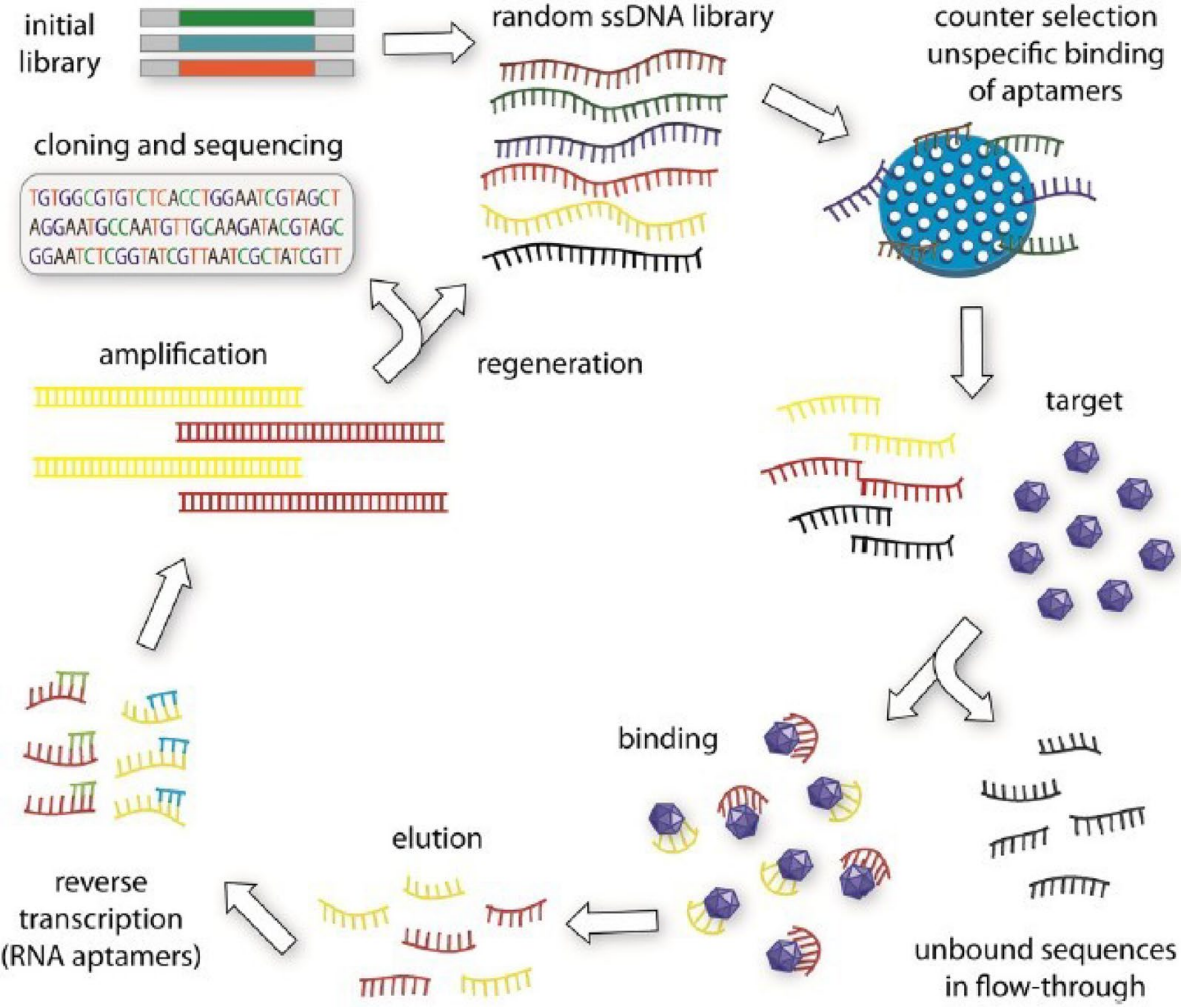
Schematic illustration of conventional SELEX. Usually, an initial random library should be synthesized for selection. Then, counter selection is always needed before incubate the library with the targets to remove non-specific binding sequences. After incubation the target-bound sequeneces are collected and amplificated by PCR method. For selecting DNA aptamers, general PCR is enough for the amplification of the secondary library. While, as for selecting RNA aptamers, reverse transcription PCR is introduced before amplification and preparation of secondary library. After several successive rounds of selection, the library is cloned and sequenced followed by evaluation and identification of the enriched aptamers (Reprinted with permission from Ref. [51]. Copyright © 2015, Elsevier)

In 1990, Tuerk and Gold [14] were the first to select two RNA sequences with the binding affinity to T4 DNA polymerase from a random RNA library in vitro, and designated the method Systematic Evolution of Ligands by EXponential enrichment (SELEX). At the same time, Ellington and Szostak [50] isolated RNA subpopulations from a large combinatorial library of 10^10^ random-sequence RNA molecules, which can specifically bind various organic dyes, and coined the term “aptamer”. Since then, the SELEX method has been widely applied for aptamer selection in vitro. As shown in Fig. 1, conventional SELEX procedures usually include: (1) random single-stranded (ss)DNA or RNA library design and synthesis (2) target binding, (3) elution, (4) PCR amplification, (5) preparation of a single-stranded oligonucleotide library, (6) multiple rounds of selection, (7) cloning and sequencing, and (8) aptamer identification [51]. As a rule, negative selection is often needed before incubation with the targets to remove non-specific sequences. However, there are differences between selection of DNA and RNA aptamers. Thus, to obtain DNA aptamers, the amplification of bound sequences is performed by a regular PCR, followed by ssDNA preparation to generate the secondary library, whereas in case of RNA aptamers, reverse transcription PCR should be used prior to the preparation of the secondary random RNA library.

The past decades have witnessed fast development of SELEX methods and applications of aptamers in various areas. Although the conventional SELEX can successfully screen aptamers in vitro, the procedures are tedious, laborious, and time-consuming. Therefore, a number of modified SELEX methods of high efficiency have been developed to select aptamers against various targets, including proteins, peptides, small chemical compounds, metal ions, bacteria, cultured cells, and even heterogeneous in vivo targets [52–60]. According to the target type, SELEX methods can be divided into purified target-based SELEX, Cell-SELEX, and in vivo SELEX [61, 62].

### Purified target-based SELEX

**Fig. 2:**
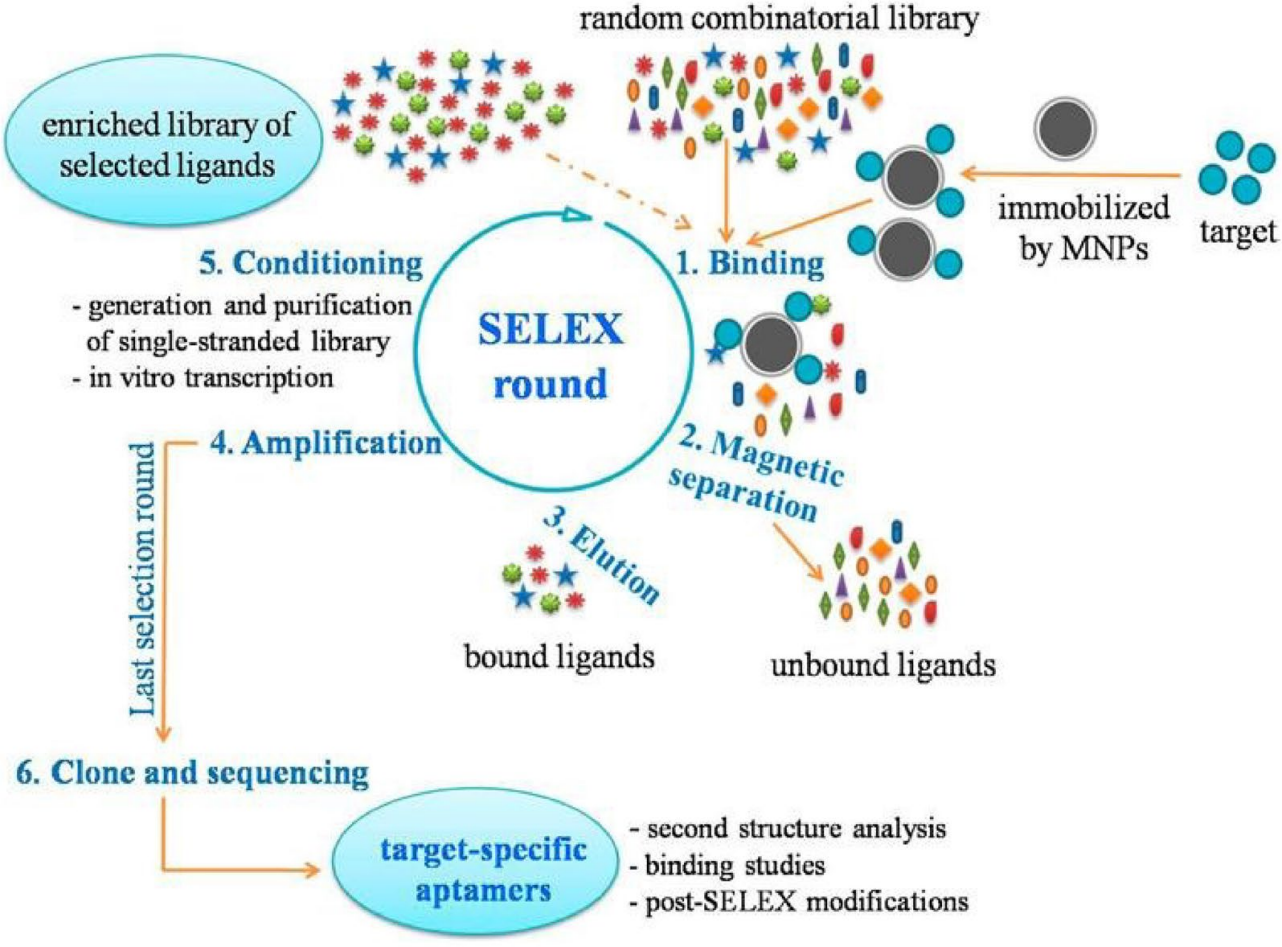
Schematic illustration of purified-target based SELEX procedures using magnetic particles as solid phase carrier for immobilization of targets (Reprinted with permission from Ref. [65]. Copyright © 2015, American Chemical Society)

Purified targets of aptamers include metal ions, small molecules, proteins, and many others [62–64]. In its general flow, SELEX of purified target-specific aptamers follow the original procedure (Fig. 2) [65], where the key step is separation of the target-bound oligonucleotides. Therefore, various solid-phase carriers are used in purified target-based SELEX, such as magnetic nanoparticles (MNPs), agarose beads, nitrocellulose membrane, and adsorptive microplates. Block et al. [66] first selected thrombin-specific ssDNA aptamers by immobilizing thrombin on a concanavalin A-agarose column. In our laboratory, Xi et al. [65] selected three DNA aptamers specifically bound to hepatitis B virus (HBV) surface antigen (HBsAg) using carboxylated MNPs for HBsAg immobilization. As a result, they generated a chemiluminescence aptasensor based on aptamer H01, which could detect HBsAg in spiked samples or clinical serum specimens with a detection limit of 0.1 ng/mL HBsAg in serum, which was lower compared to the 0.5 ng/mL limit of a clinically used ELISA. In a similar study, Huang et al. [67] obtained a DNA aptamer against HBV e antigen (HBeAg) by MNP-based SELEX and developed an aptamer-based fluorescence biosensor for highly efficient quantitative detection of up to 609 ng/mL HBeAg in serum, which could be completed within 2 min.

### Cell-SELEX

**Fig. 3:**
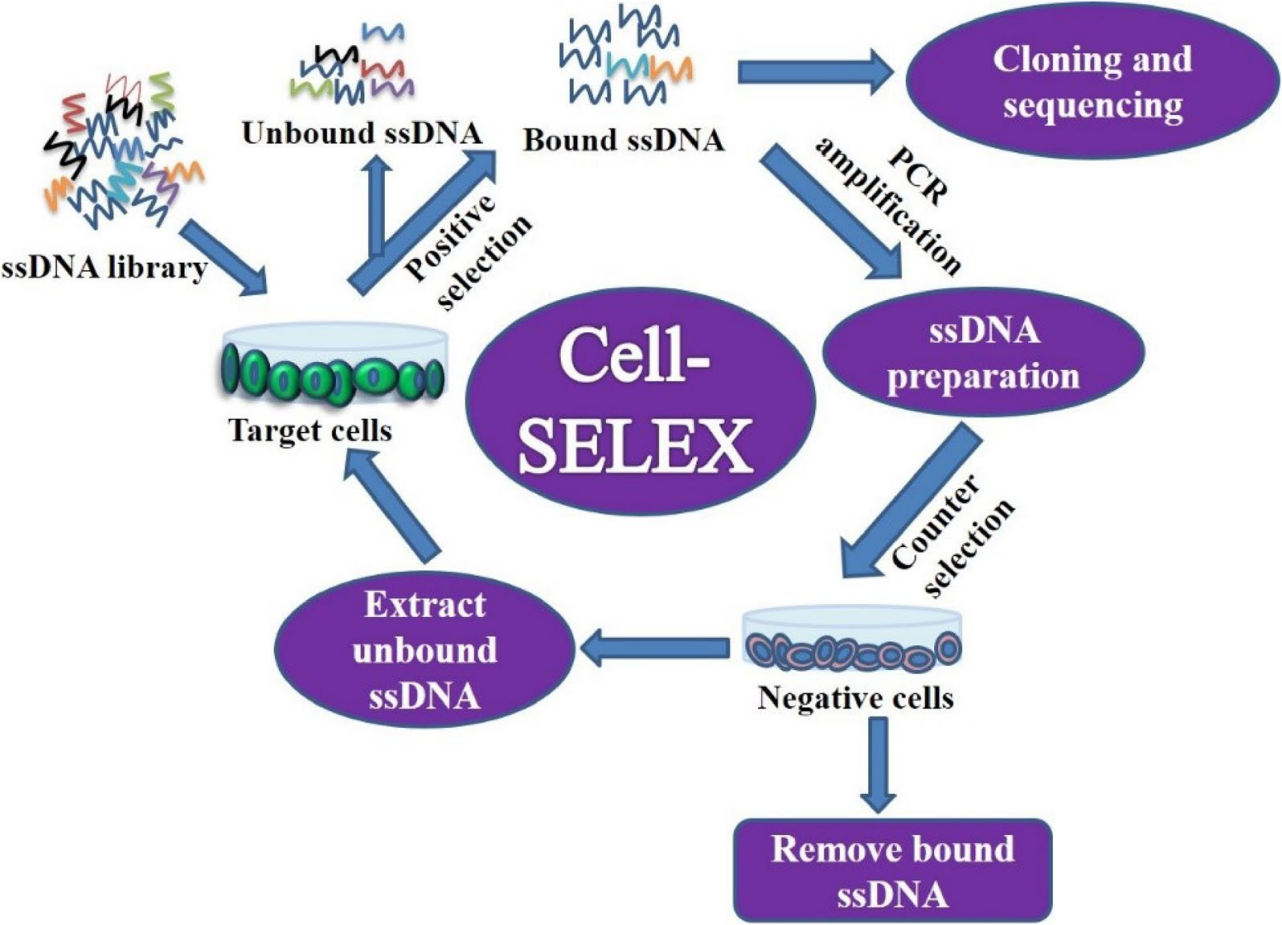
Schematic illustration of Cell-SELEX procedures

Although the achievements in aptamer selection against purified targets, especially proteins, have been significant, there are some limitations regarding the application of such aptamers, mainly due to a synthetic nature of the target proteins which differ from their native counterparts in conformation and higher order structure, and bioactivity. Consequently, the aptamers selected against artificial proteins may have much lower sensitivity in recognizing native targets. Therefore, whole living cells have been considered and successfully used for aptamer generation by the method known as Cell-SELEX [68]. The production of aptamers in Cell-SELEX generally includes both positive and negative selection. As shown in Fig. 3, 2–4 cycles of positive selection are first conducted by incubating a random-sequence oligonucleotide library with target cells to remove unbound sequences. Then, negative selection is performed by incubating the secondary library with non-target cells, after which unbound sequences are collected, applied to the target cells, eluted, amplified by PCR, and used to prepare an ssDNA secondary library for the next round of selection. Finally, when the selection process is completed, target cell-specific aptamers are PCR-amplified, cloned, and sequenced. Compared with purified target-based SELEX, Cell-SELEX can generate novel highly specific aptamers against native complex functional molecules expressed on the cell surface, without previous identification of the target. Therefore, since targets are unknown, the selected aptamers can be used to discover new molecular biomarkers [69–71].

Over the past years, hundreds of cell-specific aptamers have been obtained by Cell-SELEX. Thus, Bayrac et al. [72] successfully used a Cell-SELEX-based procedure without negative selection to generate glioblastoma multiforme-specific DNA aptamers, which showed high specificity and affinity to glioblastoma cell lines with a *K*_*d*_ in the nanomolar range. Li et al. [73] developed a panel of DNA aptamers against colon cancer SW620 cells after 14 rounds of selection using Cell-SELEX. The finally selected aptamer XL-33 showed high binding affinity (*K*_*d*_ = 0.7 nM), and its truncated counterpart XL-33-1 containing only 45 nt also displayed excellent selectivity to the target cells. Furthermore, tissue imaging indicated that XL-33-1 could distinguish between metastatic tumor tissues or lymph nodes and benign tissues with a detection rate of 81.7%. In our laboratory, we have recently successfully applied Cell-SELEX to develop a series of DNA aptamers specifically binding to cultured human hepatocellular carcinoma HepG2 cells [74]. The selected aptamers showed high binding affinity to HepG2 cells with *K*_*d*_ values ranging from 46.3 to 199.4 nM and could distinguish HepG2 cells from normal human liver cells.

### In vivo SELEX

**Fig. 4:**
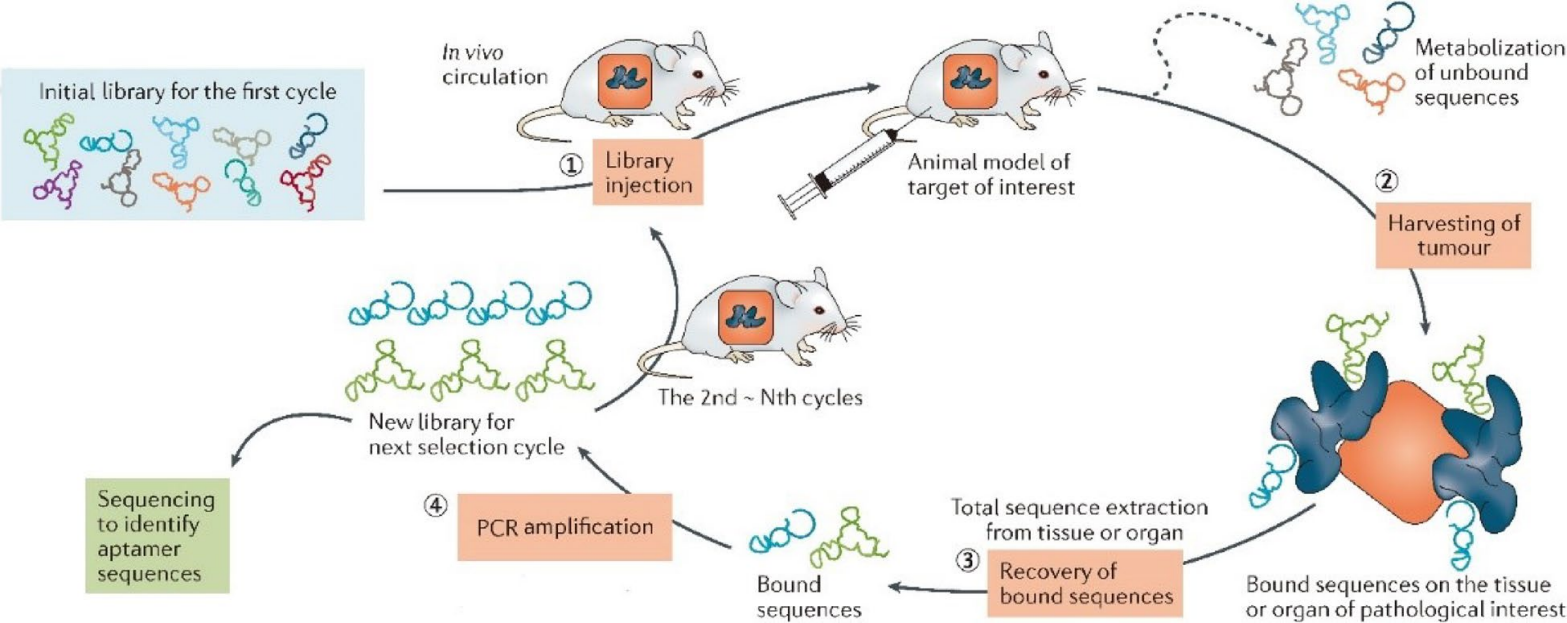
Schematic illustration of in vivo SELEX procedures (Reprinted with permission from Ref. [61]. Copyright © 2017, Nature Publishing Group)

Currently, most aptamers are selected in in vitro conditions, which provide a simple and controllable binding environment. However, considering that the ultimate goal is the application of aptamers in vivo, i.e., in a very complex physiological environment, in vitro-selected aptamers may not have sufficient stability and half-life to exert the desirable effects [75]. Therefore, generation of aptamers with physiological stability is a task of a paramount importance. In vivo SELEX is a new aptamer selection approach based on using animal models to obtain tissue- and organ-specific aptamers (Fig. 4) [61]. The detailed protocol used in in vivo SELEX is as follows: intravenous injection of a random oligonucleotide library, harvesting the tissue or organ of interest, extraction and amplification of the bound molecules, and preparation of a secondary random library for the next selection cycle. Mi et al. [76] tested a nuclease-resistant RNA aptamer against hepatic colon cancer metastases in tumor-bearing mice using in vivo SELEX, and identified the target molecule as p68, an RNA helicase upregulated in colorectal cancer. Wang et al. [77] applied in vivo SELEX to select RNA molecules specific for human non-small cell lung cancer using cultured NCI-H460 cancer cells and tumor-bearing xenograft mice, and obtained an aptamer with high specificity and affinity to both cancer cell line and mouse tumor tissues.

### Highly efficient SELEX

**Fig. 5:**
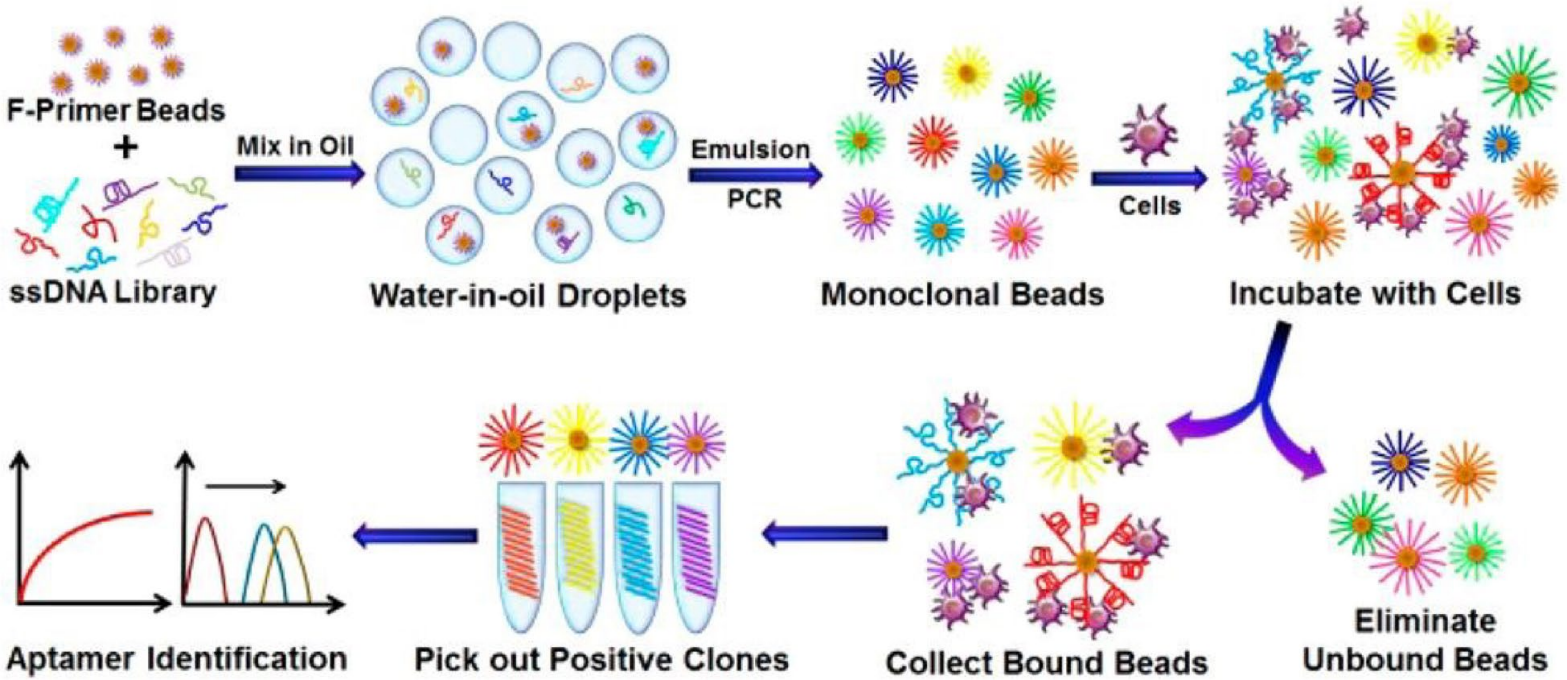
Schematic illustration of monoclonal surface display SELEX (MSD-SELEX) procedures (Reprinted with permission from Ref. [82]. Copyright © 2014, American Chemical Society)

**Fig. 6:**
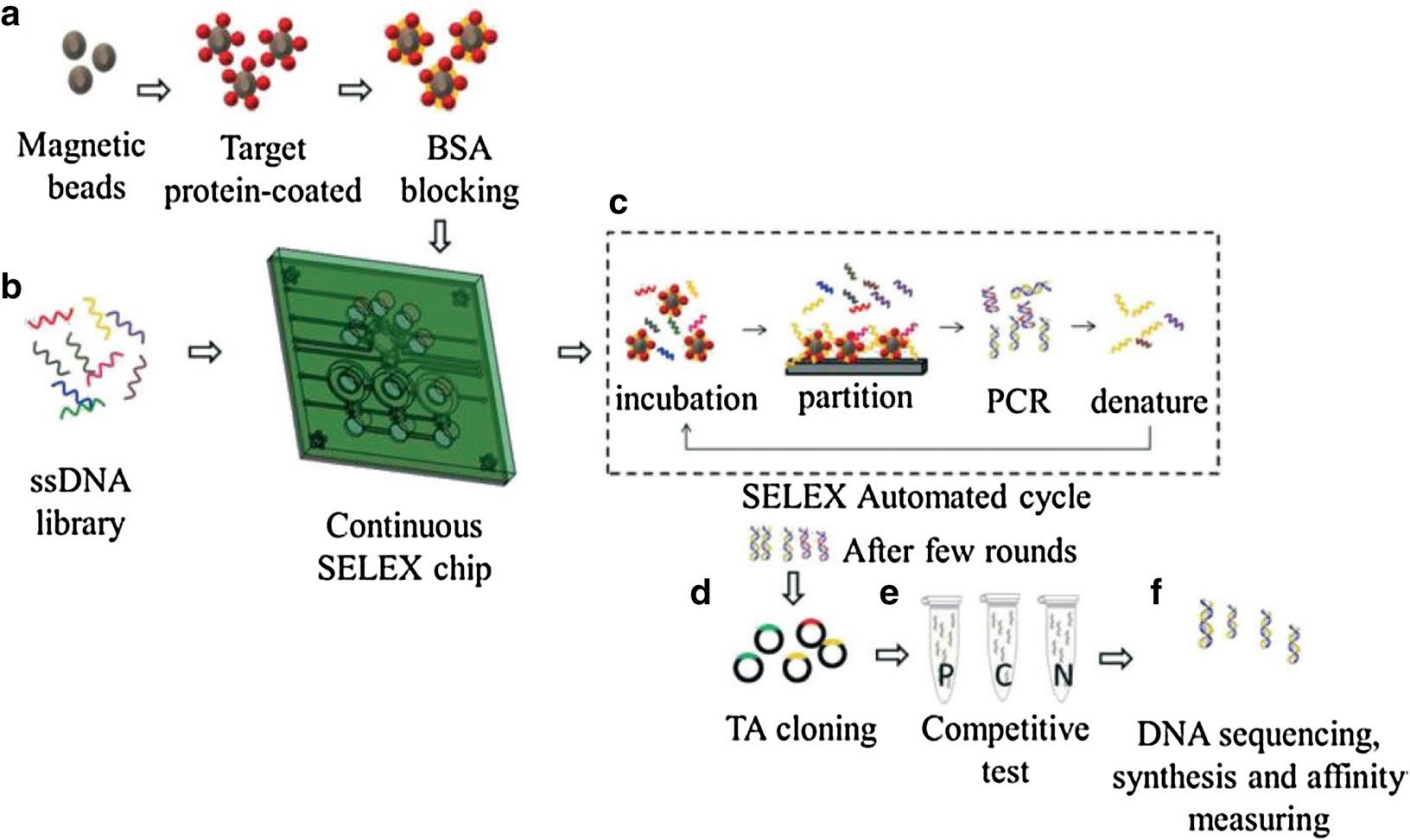
Schematic illustration of microfluidic SELEX procedures (Reprinted with permission from Ref. [85]. Copyright © 2014, Royal Society of Chemistry)

In order to improve SELEX efficiency, various approaches have been recently developed, including capillary electrophoresis SELEX (CE-SELEX) [78], microfluidic SELEX [79], high-throughput sequencing-assisted SELEX (HT-SELEX) [80, 81], monoclonal surface display SELEX (MSD-SELEX) [82], and automated SELEX [83]. Zhu et al. [82] designed a novel MSD-SELEX method for rapid and efficient selection and identification of aptamers (Fig. 5). They combined an initial library with primer-modified beads to produce a library of monoclonal DNA-displaying beads via highly parallel single-molecule emulsion PCR, which they incubated with the target. This new aptamer selection approach was afterwards successfully applied to identify high-affinity aptamers against various targets. Compared to conventional SELEX methods, the newly developed MSD-SELEX approach is simple, rapid, efficient, and cost-effective. Dong et al. [84] screened an alpha-fetoprotein-bound ssDNA aptamer using CE-SELEX technology with only four selection cycles. The aptamer could not only distinguish HepG2 cells from A549 cells by immunofluorescence imaging but also efficiently inhibited the migration and invasion of hepatocellular carcinoma cells in vivo. Moreover, Lin et al. [85] developed a microfluidic SELEX chip based on magnetic beads to select hemoglobin (Hb)- and HbA1c-specific ssDNA aptamers (Fig. 6). They coated magnetic beads with HbA1c and Hb, performed several rounds of selection and enrichment with an ssDNA library, and selected specific oligonucleotides, which were sequenced and identified. Compared with conventional SELEX methods, the developed microfluidic SELEX system dramatically decreased the incubation and partitioning time, thus simplifying the entire SELEX process. In addition, various newly developed separation and amplification technologies, including flow cytometry [86, 87], biacore surface plasmon resonance [88], atomic force microscopy [89–91], and digital PCR [92] have been integrated into SELEX to obtain aptamers with high affinity and specificity to targets.

## Applications of aptamers for breast cancer diagnostics

### Detection of breast cancer biomarkers using aptamers

**Fig. 7:**
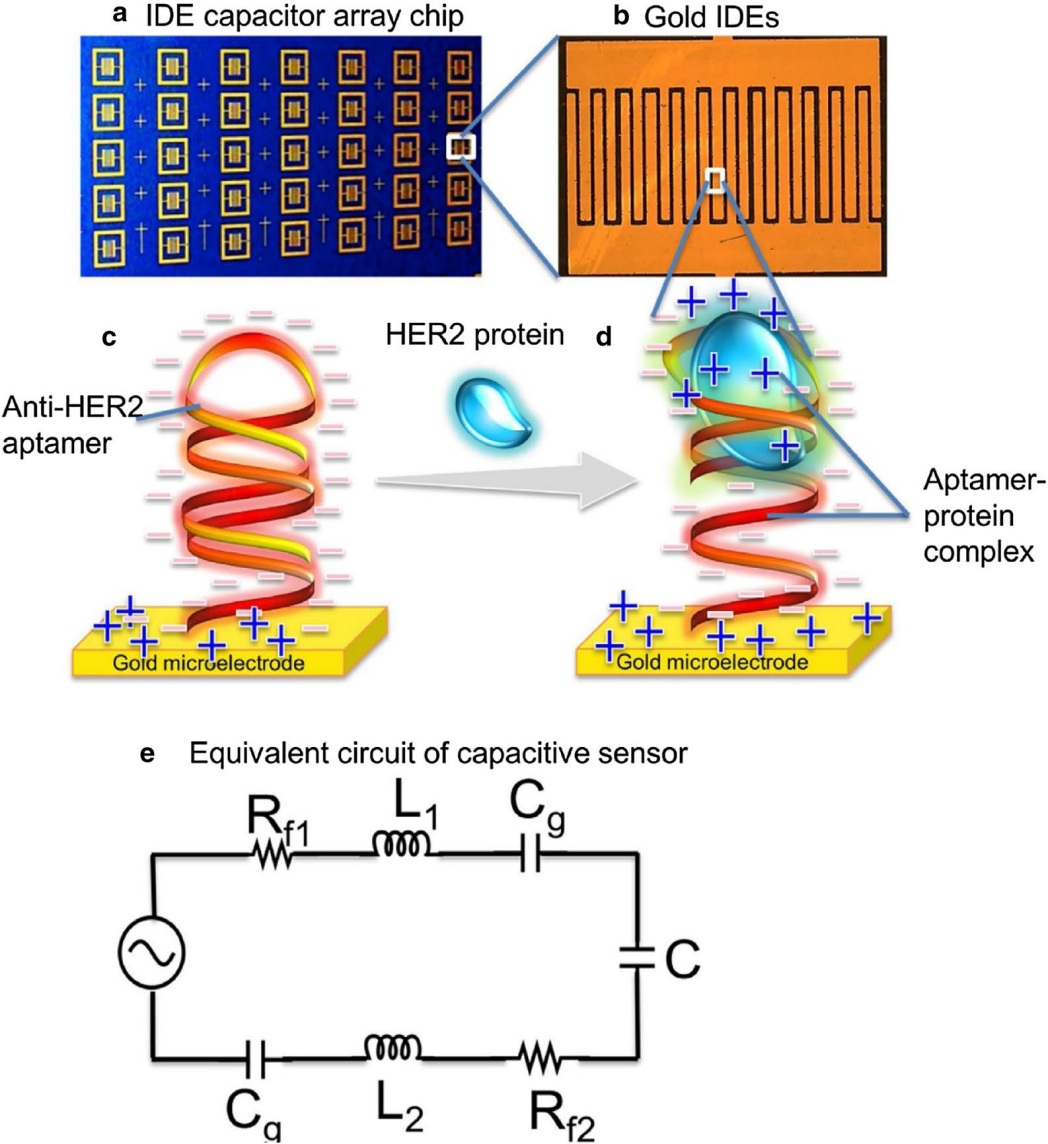
Schematic illustration of label-free capacitive aptasensor for HER2 protein detection in serum. **a** capacitor array chip, **b** capacitor, **c** gold IDE functionalized with anti-HER2 aptamer whose phosphodiester backbone contributes abundant negatively charged species on capacitors, **d** formation of aptamer-HER2 complex that induce charge distribution and thus influence specific changes in capacitance that can be measured and **e** equivalent circuit of the interdigitated capacitor sensor (Reprinted with permission from Ref. [93]. Copyright © 2015, Elsevier)

Biomarker detection plays a very important role in early diagnosis, monitoring of curative effects, and prognosis in breast cancer. Among the identified breast cancer-specific biomarkers, HER2 is one of the most important and commonly used not only for molecular classification but also for the targeted therapy of breast cancer in clinics. Niazi et al. [40] used Cell-SELEX to select an anti-HER2 ssDNA aptamer H2 which showed high binding affinity to HER2 with a *K*_*d*_ of 270 nM. As a next step, they developed a fluorescence-based magnetic microbead-carbon nanotube-H2 hybrid system for HER2 detection in vitro and demonstrated its potential for identification of the native HER2 protein in solution and biological samples. Qureshi et al. [93] have developed a label-free capacitive aptasensor by functionalizing anti-HER2 ssDNA aptamers onto interdigitated microelectrodes of the capacitor for HER2 detection in serum (Fig. 7). The aptasensor was very sensitive and could be applied to detect HER2 in human serum within a dynamic range of 0.2–2 ng/mL, demonstrating versatility and potential as a tool for early diagnosis of breast cancer.

Besides HER2, other breast cancer biomarkers were used to select aptamers for breast cancer diagnostics [94–98]. Thus, Ahirwar et al. [43] used the HT-SELEX approach to identify an ERα-specific DNA aptamer, which could be internalized by ERα-positive breast cancer cells and localize specifically in the nucleus. The aptamer was applied to reveal ERα expression in breast cancer, and the results were consistent with IHC detection of ERα in breast cancer tissues strongly and moderately positive, or negative for ERα. Li et al. [99] designed a microcantilever biosensor for label-free detection of nucleolin, a protein regulating the stability of *Bcl*-*2* mRNA in cancer cells. In their system, nucleolin-specific aptamer AS1411 was functionalized on sensor cantilevers in the microcantilever array, while reference cantilevers were modified with 6-mercapto-1-hexanol to eliminate environmental disturbances. In this aptasensor, aptamer AS1411 interaction with nucleolin in the sample would induce surface stress change, resulting in differential deflection between the sensor and reference cantilevers. The system demonstrated high sensitivity with a detection limit of about 1.0 nM.

### Detection of breast cancer cells using aptamers

**Fig. 8:**
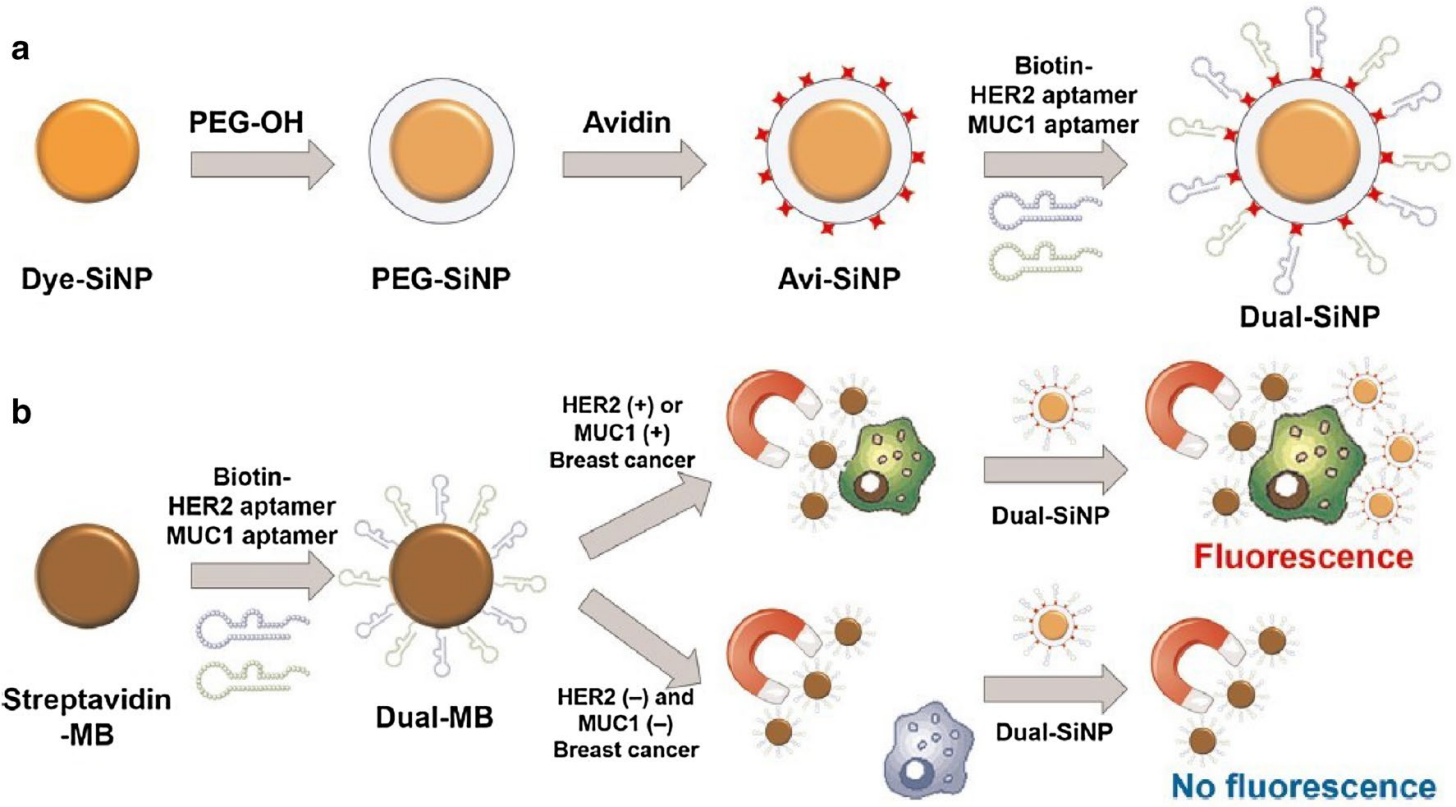
**a** Modification processes of the silica nanoparticles. Dye-SiNPs were synthesized via the reverse microemulsion technique, followed by being coated with PEG and avidin immobilization. Then, dual aptamer-functionalized dye-doped silica nanoparticles (Dual-SiNPs) were synthesized by modifying MUC1 aptamer and HER2 aptamer onto Dye-SiNPs through biotin-avidin interactions. **b** Schematic illustration of Dual-SiNPs for highly sensitive detection of MUC1(+) and HER2(+) breast cancer cells. MUC1(+) or HER2(+) breast cancer cells were selectively enriched and separated by dual aptamer-modified magnetic beads followed by being detected with Dual-SiNPs (Reprinted with permission from Ref. [106]. Copyright © 2015, Elsevier)

Identification of breast cancer cells, especially CTCs in patient serum is important for early cancer diagnosis, monitoring of treatment effects, and prognosis. Aptamers evolved from both purified target-based SELEX and Cell-SELEX provide a powerful tool for detecting breast cancer cells [100–104]. Cai et al. [105] have developed a signal-on fluorescence aptasensor using a mucin 1 (MUC1)-specific aptamer and ssDNA-sensitized luminescent terbium (TbIII) for label-free, facile, and sensitive detection of MCF-7 breast cancer cells. The principle of the method is that in the presence of MCF-7 cells the aptamer would bind to MUC1 on the cell membrane. After centrifugation, aptamer-cell conjugates would be separated from supernatant. The signal probe is then added, and in the absence of aptamer-cell conjugates, the emission of Tb^3+^ would be enhanced, whereas in their presence, the signal probe would hybridize with the aptamer and lose the ability to induce Tb^3+^ emission. The proposed aptasensor exhibited excellent sensitivity towards MCF-7 cells with a detection limit as low as 70 cells/mL. Furthermore, Jo et al. [106] synthesized dual aptamer-functionalized dye-doped silica nanoparticles based on MUC1 and HER2 aptamers for highly sensitive detection of breast cancer cells (Fig. 8). In their method, the aptamers modified on magnetic beads would capture the target cells, which would be precipitated by magnetic separation, and the dual aptamer-functionalized dye-doped silica nanoparticles are added as signal generators. Then, magnetic separation and fluorescence excitation are conducted and fluorescence signal would be detected if target cells were captured; otherwise, no fluorescence would be observed. Compared with single aptamer-based detection, this newly developed technique provides very sensitive diagnostics of breast cancer cells with a detection limit of 10 cells/mL, which is dramatically lower compared to other methods. We have recently synthesized aptamer-functionalized silver nanoclusters (AgNCs) based on a MUC1 aptamer for imaging of MCF-7 breast cancer cells [107], which could efficiently differentiate MCF-7 cells from more aggressive MDA-MB-231 breast cancer cells and A549 human lung cancer cells.

### Detection of breast cancer tissues using aptamers

Currently, IHC is still the gold standard in breast cancer detection used to confirm definite diagnosis. However, antibodies used in IHC are expensive, diffuse slowly, and always need signal amplification. In this respect, aptamers have attracted increasing attention as the means to overcome these limitations [108–111]. Thus, Gupta et al. [112] have developed a fluorescent HER2-targeting aptamer for breast cancer tissue imaging, which opened a door to aptamer application in histochemistry. Li et al. [45] used Cell-SELEX to identify a DNA aptamer LXL-1-A specifically binding to MDA-MB-231 breast cancer cells, which showed high affinity and excellent selectivity towards MDA-MB-231 cells and was utilized for breast cancer histochemistry. The results were very promising, as aptamer LXL-1-A was highly specific to breast cancer tissues, demonstrating a 76% detection rate for tumor metastasis in regional lymph nodes.

## Applications of aptamers in breast cancer therapy

Aptamers can be used not only as diagnostic tools but also as therapeutic agents, because they can, after specific binding to target proteins, either directly inhibit/activate protein function or modulate protein–protein interactions [61, 75]. Furthermore, aptamers can also be used as recognition ligands or carriers for targeted drug delivery [113, 114]. To date, although there have been a couple of aptamers clinically tested for disease treatments; only one of them has already been approved by the FDA for the treatment of all types of neovascular age-related macular degeneration [115]. The limited in vivo therapeutic applications of aptamers mainly result from the limitations of nuclease degradation, metabolism and toxicity of aptamers in the physiological environment [75, 116]. Thus, in order to decrease the degradation and improve efficiency of aptamer-based therapeutics in vivo, the original selected aptamers always need chemical modification, remoulding or conjugation with other biomaterials. In this part, we focused on the therapeutic applications of aptamers for targeted therapy of breast cancer.

### Aptamers as therapeutics

Aptamers can bind to and regulate the function of the target protein or gene expression, and may signal cells to start apoptosis or enhance their sensitivity to chemical drugs [117–121]. Bala et al. [122] selected two glutathione-binding RNA aptamers with respective *K*_*d*_ values of 41.8 and 48.9 nM, which could induce apoptosis of MCF-7 breast cancer cells through the accumulation of reactive oxygen species (ROS) and modulation of intracellular glutathione and caspase-3 activation, suggesting that glutathione-binding RNA aptamers could be developed into effective therapeutic agents for breast cancer treatment. In another study, aptamer AS1411 targeting a *Bcl*-*2* mRNA-binding protein nucleolin was examined for the ability to induce *Bcl*-*2* mRNA instability and cytotoxicity in MCF-7 and MDA-MB-231 breast cancer cells [123]. The results indicated that aptamer AS1411 could efficiently inhibit the growth of MCF-7 and MDA-MB-231 cells (but not that of normal mammary epithelial cells), decrease the half-life of *Bcl*-*2* mRNA in cancer cells, and inhibit nucleolin binding to the AU-rich element 1 in *Bcl*-*2* mRNA, promoting mRNA destabilization. These findings suggest that aptamer AS1411 is a promising molecular decoy which competes with *Bcl*-*2* mRNA for binding to cytoplasmic nucleolin in breast cancer cells, eventually inducing cell death. In another recent study, Varshney et al. [124] selected an RNA aptamer specific for the RID2 domain of human telomerase reverse transcriptase (hTERT), which could tightly and specifically bind to the hTERT peptide and regulate telomerase activity in MCF-7 breast cancer cells, suggesting this RNA aptamer a potential telomerase-blocking agent to be used for breast cancer treatment.

### Aptamer-guided drug delivery

**Fig. 9:**
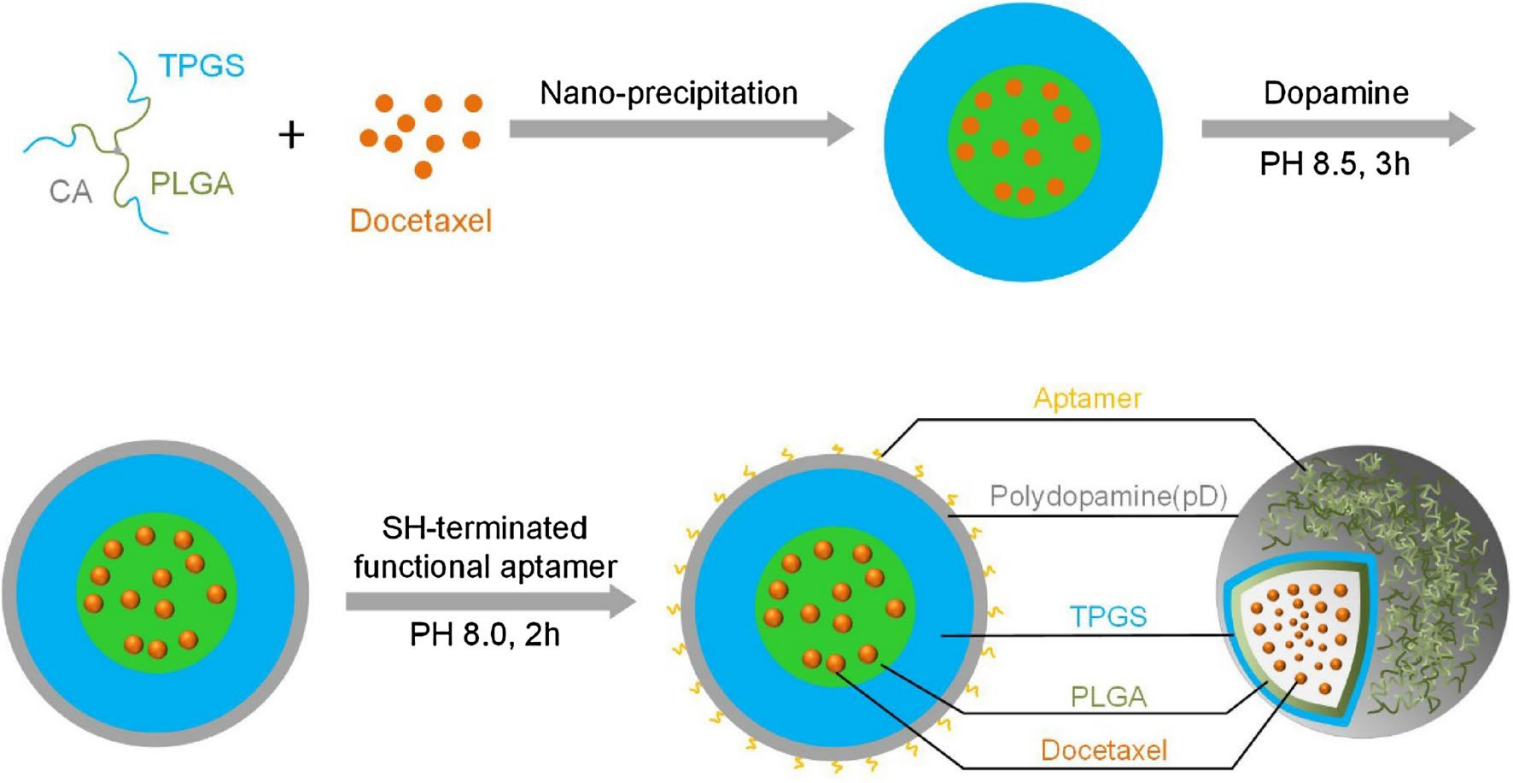
Schematic illustration of the preparation procedure of the targeted Apt-pD-DTX/NPs (Adapted with permission from Ref. [126]. Copyright © 2016, Ivyspring International Publisher)

Up to date, chemotherapy is still the major treatment approach in cancer. However, since most drugs have low selectivity towards cancer cells and can also kill normal cells, chemotherapy always causes various side effects. Therefore, targeted delivery of chemotherapeutic agents may overcome this limitation and improve their efficacy and specificity. In the past years, a number of targeted strategies have been developed for the treatment of breast cancer. Among them, aptamers can represent ideal targeting ligands with high specificity towards various cancer-related molecules; therefore, aptamer-guided drug delivery systems for breast cancer treatment have attracted considerable attention. Liu et al. [47] developed a new HER2-specific aptamer using SELEX and designed an aptamer-doxorubicin complex (Apt-Dox) by incorporating Dox into the aptamer DNA structure for targeted drug delivery to HER2-positive breast cancer cells. The results demonstrated that the aptamer-guided Dox delivery could elicit selective cytotoxic effects in HER2-positive breast cancer cells, while only weak toxicity was observed for HER2-negative cells. Moreover, Dai et al. [125] have assembled a MUC1-targeting drug delivery system using a MUC1 aptamer and a DNA tetrahedron (Td) carrying Dox within its DNA structure. As a result, the Aptamer-Td complex could selectively bind and deliver Dox to MUC1-positive breast cancer cells, demonstrating significantly higher cytotoxicity towards MUC1-positive MCF-7 breast cancer cells compared to MUC1-negative control cells in vitro (P < 0.01), suggesting a promising drug delivery system for the targeted therapy of MUC1-positive breast cancer. Most recently, Tao et al. [126] had generated novel polydopamine (pD)-modified nanoparticle-aptamer bioconjugates (Apt-pD-DTX/NPs) for in vivo targeted therapy of breast cancer (Fig. 9). Both in vitro cell experiments and in vivo animal studies demonstrated that the Apt-pD-DTX/NPs could enhance targeting efficiency and therapeutic effects, reduce the adverse effects of anti-tumor drugs, and improve survival quality during treatment, indicating the Apt-pD-DTX/NPs a potentially qualified drug delivery system for breast cancer therapy.

**Fig. 10:**
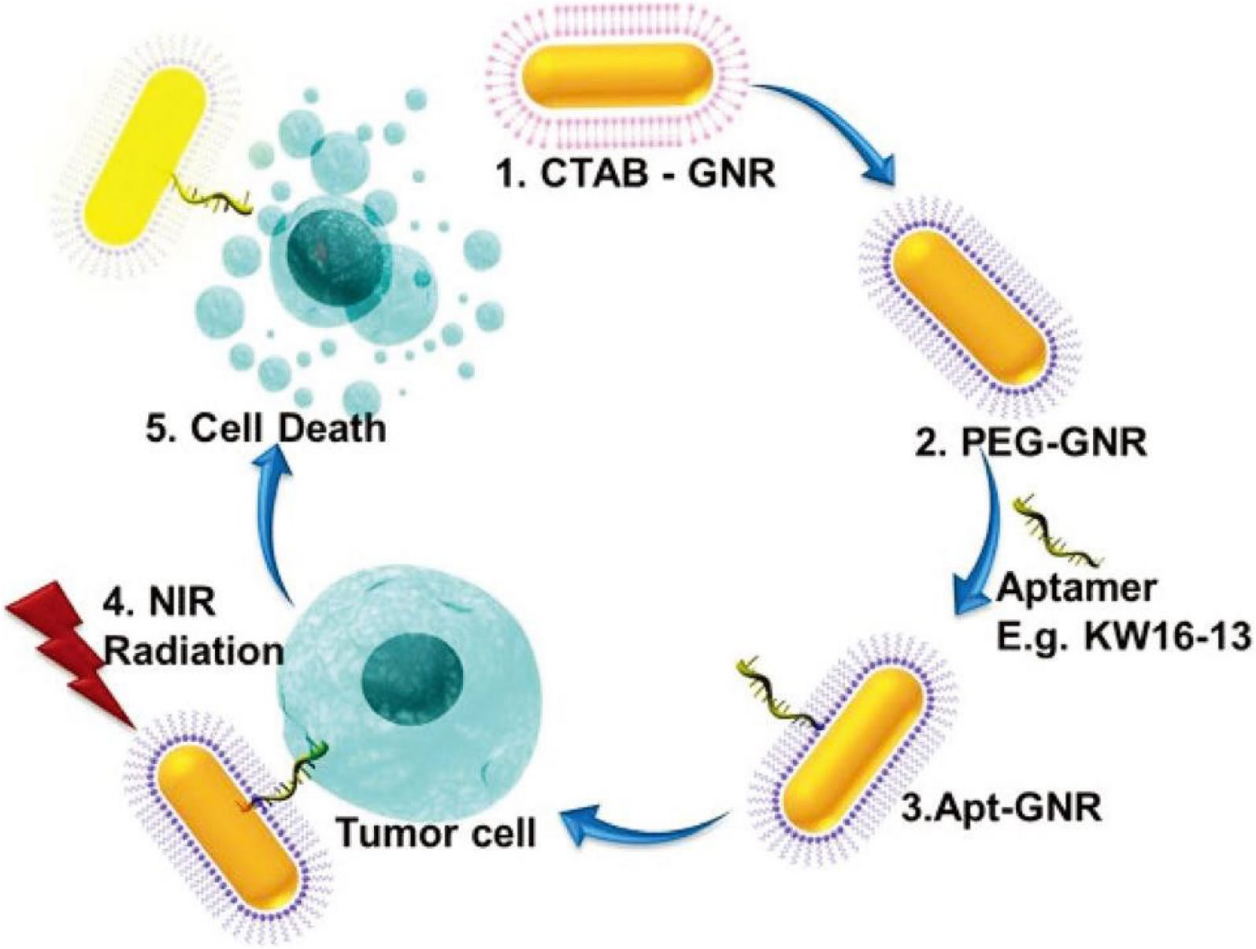
Schematic illustration of the synthesis and use of aptamer functionalized gold nanorods (Apt-GNRs) for targeted photothermal therapy of breast cancer. First, cetyl trimethylammonium bromide-GNRs (CTAB-GNRs) were synthesized via a seed-mediated approach (1). Secondly, the CTAB was replaced by PEG (2) for functionalizing aptamer (3). Then, the Apt-GNRs were incubated with the targeted breast cancer cells followed by photothermal ablation in near infrared range (4), which eventually caused targeted cell death specifically (5) (Reprinted with permission from Ref. [130]. Copyright © 2015, Royal Society of Chemistry)

Aptamer-nanomaterial conjugates have also been extensively studied in relation to cancer treatment [127]. During the past years, a series of aptamer-nanomaterial conjugates based targeted therapies of breast cancer have also been widely investigated, showing great potential for clinical transformation. Beqa et al. [128] designed a novel hybrid nanomaterial consisting of gold nano popcorn-attached single wall carbon nanotubes for targeted photothermal treatment of breast cancer. The results demonstrated that the targeted SK-BR-3 human breast cancer cells could be killed within 10 min using laser irradiation at 785 nm with 1.5 W/cm^2^ power. In another study, researchers took advantage of gold nanorods (GNRs), which have strong broad-band longitudinal surface plasmon absorption in the range of 600–1100 nm [129], and synthesized a novel DNA aptamer-tethered GNRs for efficient photothermal therapy of breast cancer [130] (Fig. 10). They used Cell-SELEX to obtain a novel DNA aptamer KW16-13 specific for human breast ductal carcinoma MCF10CA1h cells, which was then conjugated to GNRs and applied for targeted photothermal therapy. The results indicated that the aptamer-GNR conjugates had more than 70-fold specificity to MCF10CA1h tumor cells over normal cells and could be specifically internalized by and cause death in 96% of tumor cells after near-infrared light irradiation. Malik et al. [131] conjugated aptamer AS1411 to gold nanospheres (GNSs) and synthesized AS1411-linked GNSs for targeted therapy of human breast cancer. AS1411-GNSs showed high stability both in solutions and serum, and could be easily internalized by target cells in higher doses and cause increased anti-proliferative/cytotoxic effects compared with pure AS1411 or GNSs modified with control oligonucleotides. Furthermore, the administration of AS1411-GNSs in vivo could dramatically inhibit the growth of xenografted tumors in mice without any toxic side effects, suggesting AS1411-GNSs a promising drug candidate for clinical application in breast cancer treatment.

Besides chemical drugs and nanomaterials, aptamers can also deliver small interfering (si)RNA for gene therapy. Thiel et al. [132] covalently linked a HER2-specific RNA aptamer to siRNA targeting the anti-apoptotic *Bcl*-*2* gene for use in HER2-positive breast cancer. The aptamer-siRNA conjugates could be selectively taken by HER2-positive breast cancer cells and silence *Bcl*-*2* expression, enhancing the sensitivity of HER2-positive breast cancer cells to chemotherapy. Wang et al. [133] designed an aptamer-siRNA-based system targeting negative apoptosis regulator survivin, which is overexpressed in cancer stem cells of Dox-resistant breast cancer tumors and plays a very important role in drug resistance. They used an RNA aptamer specific for epithelial cell adhesion molecule (EpCAM) and a Dicer substrate survivin siRNA for the treatment of Dox-resistant breast cancer in vivo. The results demonstrated that the aptamer-guided and CSC-targeted in vivo RNAi could deliver a high siRNA dose to breast cancer stem cells in xenograft tumours, which resulted in silencing of survivin and the increase in cancer cell chemo-sensitivity, and eventually suppressed tumour growth, and prolonged the survival of mice bearing xenograft tumours.

## Conclusions and future perspectives

In recent years, aptamers have become a focus of research related to diagnostics and treatment of breast cancer because of their advantages over conventional cancer-inhibiting antibodies, such as high target specificity and affinity, good stability, non-immunogenicity, easy modification, and low production cost. In the past decades, a number of aptamers selected against breast cancer biomarkers and/or cells showed great potential for improving diagnostic and treatment rates in breast cancer. In the first part of the review, we discussed and compared various aptamer selection methods, including purified-target based SELEX, Cell-SELEX, in vivo SELEX, and high-efficiency SELEX techniques. Although aptamers evolved via purified target-based SELEX and Cell-SELEX are very promising as detection agents in vitro, they are very easily degraded in physiological environment together with bad efficiency. Therefore, selecting aptamers with good stability and efficiency in physiological environment is very important. Thus, in vivo SELEX was subsequently developed for selection of novel aptamers stable and effective enough in physiological conditions, which can be combined with chemical modification to further enhance the stability and therapeutic efficiency of selected aptamers in vivo. Although the above three SELEX methods can screen aptamers successfully, the reproducibility is not very clear and the selection efficiency is very low. Nowadays, a series of highly efficient SELEX methods have also been developed, but they have the worse stability and reproducibility in comparison with the traditional SELEX methods. Finally, some highly efficient SELEX methods need sophisticated equipment, which is difficult to meet in most laboratories in the world.

In the second part, we specifically focused on aptamer-based detection of breast cancer biomarkers, cells, and tissues, and targeted therapy. Aptamers selected using breast cancer biomarkers and/or cells can be developed into a series of aptasensors for fast, sensitive, and highly specific detection of breast cancer, representing promising candidate agents for early cancer diagnosis. Furthermore, aptamers can be utilized as therapeutics, drug carriers, or recognition ligands for targeted drug delivery in breast cancer treatment. Up to date, various aptamer-based drug delivery systems have been developed; among them, aptamer-guided nanomaterials or nanomaterial-based carriers are the most popular as they can enhance the efficiency of both drug loading and release. In these systems, aptamers always serve as specific recognition ligands, while nanomaterials and nanomaterial-based carriers loaded with chemical drugs act as therapeutic agents. However, only one aptamer-based drug has been approved by the FDA, although there are several aptamer-based drugs being tested in clinic. The main reasons are the instability and nonspecific binding in vivo. Studies demonstrated that aptamers selected in vitro are very prone to nuclease in serum and can be easily degraded, resulting in instability in vivo [116]. Besides, the negative charge of aptamers can make them nonspecifically bind to molecules or ions with positive charge, when it comes to in vivo applications. Moreover, due to the small size and small molecular mass, aptamers can be filtrated rapidly by kidney and nonspecifically accumulated in non-target organs such as liver and spleen, which may lead to adverse effects and reduce therapeutic efficiency. Although the toxicity of aptamers is demonstrated to be low in vitro, their in vivo toxicity are still controversial, which needs more detailed investigation. Besides, the nonspecific binding and accumulation may cause serious immune activation and polyanionic effects. All the above problems and challenges of aptamers eventually decrease the physiological stability and efficiency of aptamers, which dramatically limits their in vivo and clinic applications.

To advance aptamer applications in clinic medicine, the above problems and challenges should be thoroughly solved. On one hand, high efficient SELEX methods with good stability, reproducibility and low cost-effectiveness ratio should be developed for screening aptamers with high specificity, binding affinity, physiologic stability, and low adverse effects in vivo. On the other hand, although various versatile nanomaterials with properties such as monodispersity, good biocompatibility, low toxicity, high entrapment efficiency, and response-mediated release have been synthesized and investigated [134–136], scarcely are they approved for clinic medicine. Therefore, designing and synthesizing new nanomaterials meeting the requirement of clinical utilization should be addressed, either. Finally, the in vivo stability of aptamer-based therapeutics should be increased and immunogenicity monitored should be brought to a minimum. For improving the stability of aptamers in physiological environment, chemical modifications are usually conducted with groups such as fluoro, amino, O-methyl and so on [137–139]. Besides, modified nucleotides have also been applied to aptamer selection, which plays a significant role in stabilizing oligonucleotides against nuclease-mediated degradation in vivo. Another important factor concerned with the stability of aptamers in vivo is renal filtration. The molecular mass of aptamers always ranges from 5 to 15 kDa, which are very prone to renal filtration, despite their good resistance to nuclease-mediated degradation in vivo. However, the incorporation of nanomaterials will dramatically reduce renal filtration rates of aptamers. Therefore, more aptamer-nanomaterial conjugates should be developed to decrease the renal filtration and improve therapeutic efficiency of aptamers. Finally, spiegelmers (l-aptamers), which are the enantiomers of wild-type RNA aptamers (d-aptamers), have also been widely investigated for therapeutic applications due to their excellent resistant to nuclease degradation in vivo [140–142]. Because l-aptamer sequences are intrinsically insensitive to nucleases that can not be degraded, thus possessing better biostability than d-aptamer sequences in vivo. However, to obtain spiegelmers, enantiomeric targets are always needed and only some correlated molecules could be applied, which limits their applications.

## Abbreviations

SELEX: Systematic Evolution of Ligands by Exponential enrichment
ER: estrogen receptor
PR: progestrone receptor
HER2: human epidermal growth factor receptor 2
IHC: immunohistochemistry
CTCs: circulating tumor cells
MNPs: magnetic nanoparticles
HBV: hepatitis B virus
HBsAg: hepatitis B virus surface antigen
HBeAg: hepatitis B virus e antigen
ssDNA: single-stranded DNA
CE-SELEX: capillary electrophoresis SELEX
HT-SELEX: high-throughput sequencing-assisted SELEX
MSD-SELEX: monoclonal surface display SELEX
Hb: hemoglobin
AgNCs: silver nanoclusters
ROS: reactive oxygen species
hTERT: human telomerase reverse transcriptase
Apt-Dox: aptamer-doxorubicin complex
Apt-pD-DTX/NPs: polydopamine (pD)-modified NP-aptamer bioconjugates
GNRs: gold nanorods
GNSs: gold nanospheres
siRNA: small interfering RNA
EpCAM: epithelial cell adhesion molecule
